# Resistomycin Suppresses Prostate Cancer Cell Growth by Instigating Oxidative Stress, Mitochondrial Apoptosis, and Cell Cycle Arrest

**DOI:** 10.3390/molecules28237871

**Published:** 2023-11-30

**Authors:** Abeer S. Aloufi, Ola A. Habotta, Mohamed S. Abdelfattah, Marina N. Habib, Mohamed M. Omran, Sally A. Ali, Ahmed E. Abdel Moneim, Shereen M. Korany, Aisha M. Alrajhi

**Affiliations:** 1 Department of Biology, College of Science, Princess Nourah bint Abdulrahman University, P.O. Box 84428, Riyadh 11671, Saudi Arabia; asaloufi@pnu.edu.sa (A.S.A.); smkorany@pnu.edu.sa (S.M.K.); amalrajhi@pnu.edu.sa (A.M.A.); 2 Department of Forensic Medicine and Toxicology, Faculty of Veterinary Medicine, Mansoura University, Mansoura 35516, Egypt; ola_ali@mans.edu.eg; 3 Chemistry Department, Faculty of Science, Helwan University, Cairo 11795, Egypt; mabdelfattah@science.helwan.edu.eg (M.S.A.); marinanabil2104@gmail.com (M.N.H.);; 4 Botany and Microbiology Department, Faculty of Science, Helwan University, Cairo 11795, Egypt; sally_ali@science.helwan.edu.eg; 5 Zoology and Entomology Department, Faculty of Science, Helwan University, Cairo 11795, Egypt

**Keywords:** resistomycin, oxidative stress, prostate cancer, apoptosis, 5-FU

## Abstract

Globally, prostate cancer is among the most threatening and leading causes of death in men. This study, therefore, aimed to search for an ideal antitumor strategy with high efficacy, low drug resistance, and no or few adverse effects. Resistomycin is a natural antibiotic derived from marine actinomycetes, and it possesses various biological activities. Prostate cancer cells (PC3) were treated with resistomycin (IC_12_._5_: 0.65 or IC_25_: 1.3 µg/mL) or 5-fluorouracil (5-FU; IC_25_: 7 µg/mL) for 24 h. MTT assay and flow cytometry were utilized to assess cell viability and apoptosis. Oxidative stress, apoptotic-related markers, and cell cycle were also assessed. The results revealed that the IC_50_ of resistomycin and 5-FU on PC3 cells were 2.63 µg/mL and 14.44 µg/mL, respectively. Furthermore, treated cells with the high dose of resistomycin showed an increased number of apoptotic cells compared to those treated with the lower dose. Remarkable induction of reactive oxygen species generation and lactate dehydrogenase (LDH) leakage with high malondialdehyde (MDA), carbonyl protein (CP), and 8-hydroxyguanosine (8-OHdG) contents were observed in resistomycin-treated cells. In addition, marked declines in glutathione (GSH), superoxide dismutase (SOD), catalase (CAT), and glutathione peroxidase (GPx) in PC3 cells subjected to resistomycin therapy were observed. Resistomycin triggered observable cell apoptosis by increasing Bax, caspase-3, and cytosolic cytochrome c levels and decreasing Bcl-2 levels. In addition, notable downregulation of proliferating cell nuclear antigen (PCNA) and cyclin D1 was observed in resistomycin-treated cancerous cells. According to this evaluation, the antitumor potential of resistomycin, in a concentration-dependent manner, in prostate cancer cells was achieved by triggering oxidative stress, mitochondrial apoptosis, and cell cycle arrest in cancer cells. In conclusion, our investigation suggests that resistomycin can be considered a starting point for developing new chemotherapeutic agents for human prostate cancer.

## 1. Introduction

Worldwide, one of the most prevalent tumors ranked as the second cause of mortality in men is prostate cancer (PC). It develops in men mostly in old age (above 65 years old) [1]. According to the latest surveys, it is expected that about 170 thousand patients and 500 thousand deaths from PC will be recorded by 2030 [2]. The primary cause of the development of PC is thought to be the enlarged gland, which results in decreased urinary outflow volume [3]. In addition, there are several risk factors that may be involved in this malignancy, such as family history, aging, hormonal disturbance, race, unbalanced diet, overweightness, inflammatory reaction, and other environmental factors [4,5]. It was also reported that exposure to toxic xenobiotics, such as bisphenol A, alcohols, smoking, carbon tetrachloride, and heavy metals may enhance the oxidative stress that fastens the spread of the problem [3]. The resistance of cancerous cells to treatment is a major obstacle in the management of PC, and metastasis to other organs is also a common complication that worsens the situation [6].

The treatment strategies for localized PC can be performed through radical prostatectomy, anti-testosterone therapy, radiation, or a combination. Nevertheless, about 30% of cases were observed to develop advanced PC [7]. The therapeutic approach for advanced PC cases depends on the administration of FDA-approved agents, including cabazitaxel, docetaxel, radium-223, abiraterone, enzalutamide, and sipuleucel-T [8]. Unfortunately, these chemical entities evoke serious adverse effects, and the cancer cells develop resistance to the chemotherapeutic agent [9]. Additionally, 5-fluorouracil (5-FU) is a commonly used chemotherapeutic agent for treating cancer, particularly in cases of breast and prostate cancer. However, the most common adverse effects of the drug are still nausea, vomiting, mucositis, stomatitis, and diarrhea [10]. Therefore, major concerns have been raised to focus on the drug’s safety, efficacy, selectivity, and cost.

The marine environment has a great biodiversity of microbial organisms whose secondary metabolites possess promising therapeutic activities [11,12,13]. Among these microbes, *Actinomycetes*, especially *Streptomyces* spp., are enriched with bioactive compounds with antibacterial, antiviral, anti-inflammatory, antimalarial, or anticancer activities [14]. Marine actinomycetes are extensively distributed in marine ecosystems, including in water, sand, deep-sea sediments, and sponges [13]. In Egypt, the Red Sea and the Mediterranean Sea are marine habitats with great microbial biodiversity with unique types and structures [14]. Resistomycin (also known as Heliomycin) is a unique quinone-related natural antibiotic that is a secondary metabolite isolated from *Streptomyces resistomycificus* [15]. As per the literature, it exhibited different activities, including anticancer potency [15,16,17]. In addition, strong cytotoxicity was observed in resistomycin-treated HepG2 cells together with a lower cytotoxic effect in normal human kidney and hepatocyte cells [18]. Outstandingly, heliomycin and some of its derivatives inhibited tumor growth on a panel of cancer cell lines, including the drug-resistant ones [17].

Quinones are essential for many biological processes, such as electron transport and oxidative phosphorylation. Medications with quinone moieties have a wide range of pharmacological uses, including anticoagulant, anticancer, and antimalarial properties. In this regard, it has been observed that two common ortho-quinones, tanshinone IIA and beta-lapachone, which are generated from natural products, exhibit a variety of anticancer actions in malignancies, such as colorectal, breast, and cervical cancers [19]. Other quinone-related derivatives, such as 1-hydroxy-1-norresistomycin and resistoflavine, were found to have substantial cytotoxic activity in vitro against gastric adenocarcinoma (HMO2) and hepatic carcinoma (HepG2) cell lines, but their mechanisms of action require more study [20]. Furthermore, doxorubicin is a quinone medication that belongs to the anthracycline class, and it is used clinically to treat solid tumors and acute lymphoblastic and myeloblastic leukaemia [21]. Quinone’s ability to bind covalently to DNA and microsomal proteins, as well as to enhance the production of reactive oxygen species, is what gives it its anticancer properties [21].

To our knowledge, the anticancer efficacy of resistomycin has not been investigated on PC cells. Therefore, in the current study, the in vitro response of PC3 to resistomycin therapy was assessed in comparison with 5-FU and the mechanisms underlying its action. The implication of reactive radicals and oxidative stress along with mitochondrial apoptotic pathways were evaluated. Moreover, the purpose of this study was to compare the efficacy of resistomycin with that of 5-FU for treating prostate cancer. Our results offer new insights for the application of marine metabolites as a promising therapeutic strategy in cancer management.

## 2. Results

**Figure 1:**
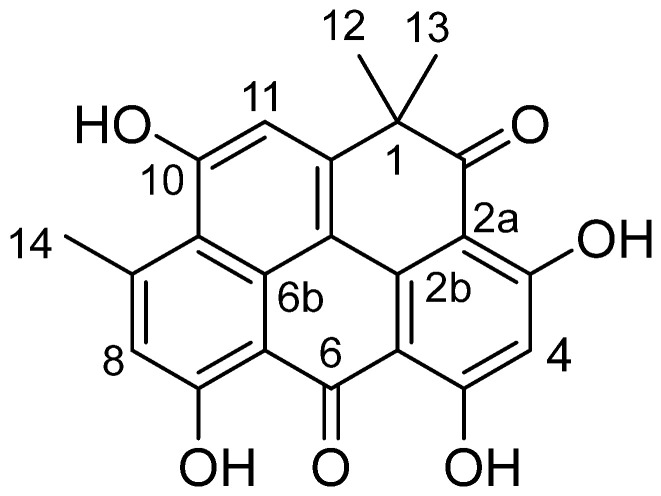
Structural formula of resistomycin (**1**).

Compound **1** was isolated as an orange solid with a molecular weight of 376 Daltons based on the negative mode of electrospray ionization mass spectrometry (ESI-MS) technique (Figure S1). The UV spectra of compound **1** exhibited absorption peaks at *λ*_max_ 455, 321, and 266 nm, suggesting the existence of a *peri*-hydroxy quinone group (Figure S2). As shown in Figure S3, the ^1^H NMR spectrum of compound **1** exhibited the presence of four hydroxyl groups that are chelated. These hydroxyl groups were observed at chemical shift values of δ_H_ 14.55, 14.36, 14.07, and 11.72 ppm. Additionally, three singlet signals corresponding to aromatic protons were detected at chemical shift values of δ_H_ 7.21, 7.01, and 6.31 ppm. The spectra also exhibited a single methyl signal at a chemical shift of δ_H_ 2.90 ppm, as well as two magnetically equivalent methyl groups that appeared as a singlet at a chemical shift of δ_H_ 1.56 ppm. The ^13^C NMR spectrum of compound **1**, as depicted in Figure S4, exhibited distinct peaks corresponding to two carbonyl groups at chemical shifts of δ_C_ 204.9 and 183.5. Additionally, the spectrum displayed signals for four aromatic carbons with oxygen substituents, observed at δ_C_ 170.7, 170.5, 167.6, and 162.1. Furthermore, twelve more aromatic carbons were identified in the spectrum, ranging in chemical shifts from δ_C_ 152.7 to 99.4. The spectrum also revealed the presence of a single aliphatic carbon (δ_C_ 46.1), two methyl groups (δ_C_ 28.9), and one aromatic methyl (δ_C_ 25.5). By searching in SciFinder, compound **1** was recognized as resistomycin (Figure 1). This was confirmed by comparing its NMR data with the values reported in the relevant literature [22].

### 2.1. Antiproliferative Activity of Resistomycin and Its Half Maximal Inhibitory Concentration (IC_50_)

**Figure 2:**
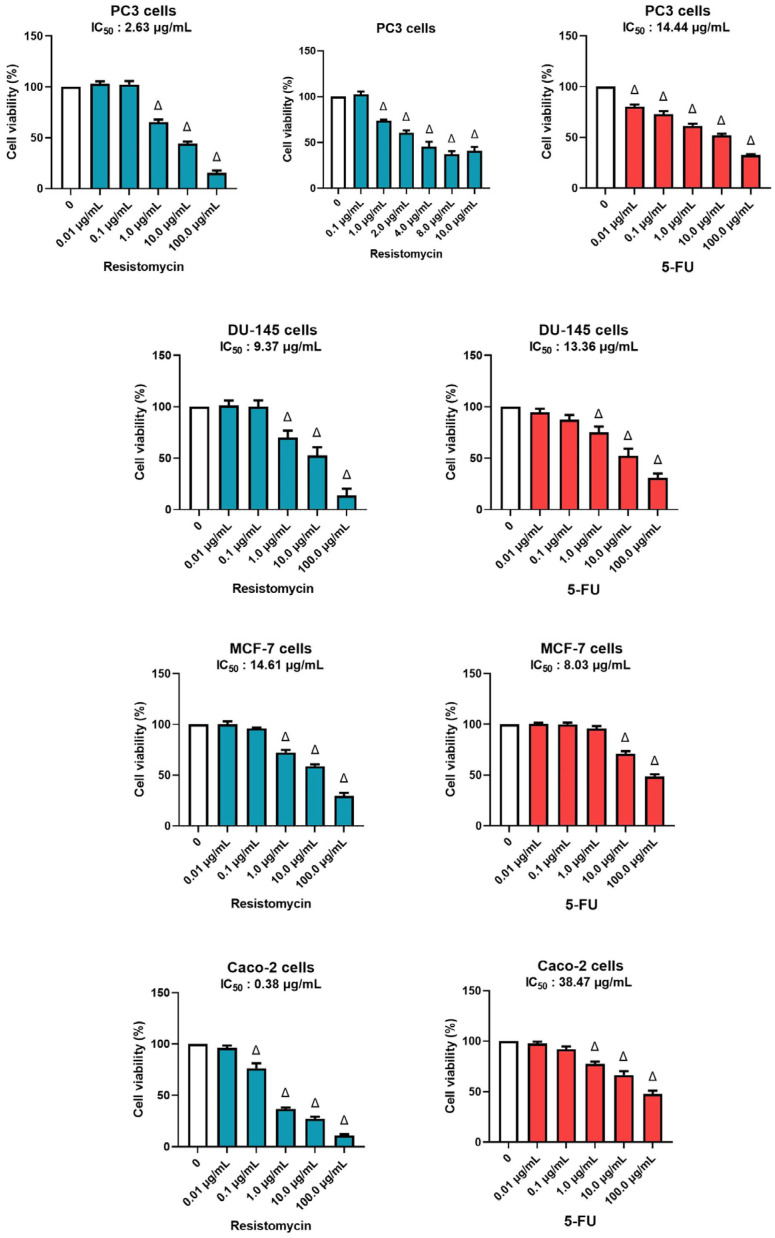
Effects of different concentrations of resistomycin or 5-FU treatment (24 h) on PC3, DU-145, MCF-7, and Caco-2 cell lines. The cell viability rate was indicated as a % of control cells without the test sample. Values represent the mean of three experiments ± SD. ∆: significant with respect to the control (*p* ˂ 0.05).

The cytotoxic potency of resistomycin on four cancer cell lines was tested with different concentrations in comparison with 5-FU (a chemotherapeutic agent) as a positive control (Figure 2). Regarding the response on PC3, a notable decrease was observed in cell viability in resistomycin-treated groups in relation with the untreated control (0 μg/mL). The IC_50_ values for resistomycin and 5-FU on PC3 cells were 2.63 μg/mL, and 14.44 μg/mL, correspondingly. The regression curve and the related equations are presented as supplementary data (Figures S5 and S6). The IC_50_ values for resistomycin and 5-FU on another prostate cancer cell line, DU-145 cells, were 9.37 μg/mL and 13.36 μg/mL, respectively. Moreover, resistomycin, at different concentrations, was assayed against Caco-2 cells. Our study unveiled that the IC_50_ values for resistomycin and 5-FU in Caco-2 cells were 0.38 μg/mL and 38.74 μg/mL, correspondingly. Further, the IC_50_ values for resistomycin and 5-FU in MCF-7 cells were 14.61 μg/mL and 8.03 μg/mL. Based on these findings, the cytotoxicity of resistomycin was higher than that of 5-FU in all tested cell lines, respectively.

### 2.2. Resistomycin Increased the LDH Release in PC3 Cells

**Figure 3:**
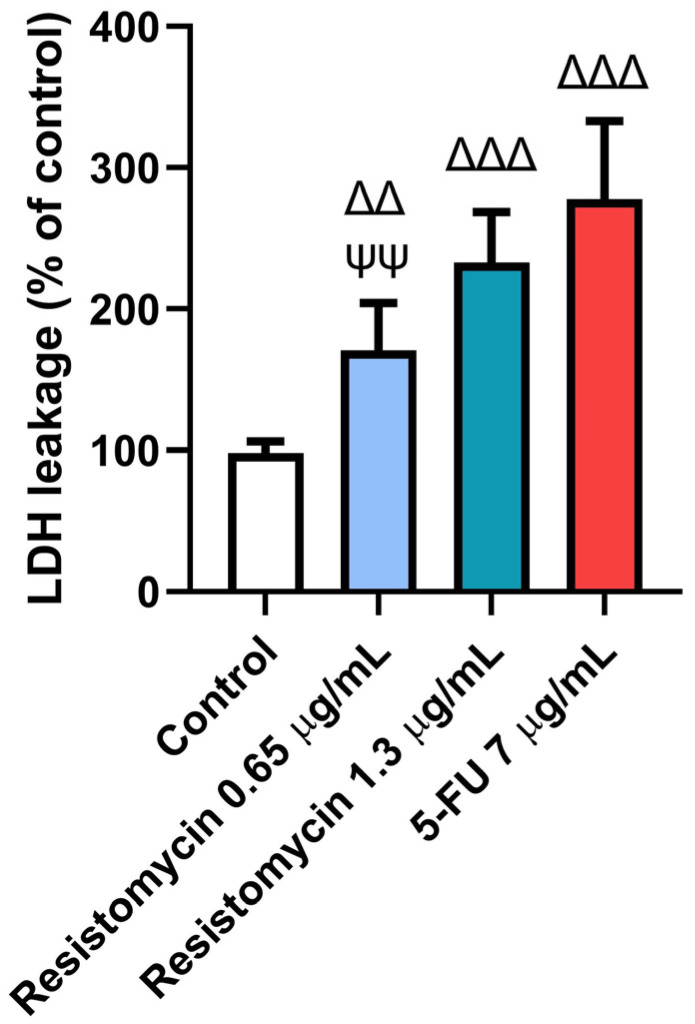
Effects of resistomycin (0.65 or 1.3 µg/mL) or 5-FU treatment (24 h) on LDH leakage in PC3 cells. Values represent the mean of three experiments ± SD. ∆∆: significant with respect to the control (*p* ˂ 0.01). ∆∆∆: significant with respect to the control (*p* ˂ 0.001). ΨΨ: significant with respect to the 5-FU (*p* ˂ 0.01).

In order to confirm the cytotoxic effect of resistomycin, the lactate dehydrogenase (LDH) leakage was measured in treated PC3 cells. The results of LDH assays indicated that 0.65 μg/mL and 1.3 μg/mL resistomycin treatments increased (*p* < 0.05) the damage of PC3 cells in comparison with the controls. Remarkably, the higher concentration of resistomycin induced a higher cytotoxic effect (*p* < 0.05) on the tested cancer cells than that of the lower one. No significant change was noticed in the percentage of LDH activity between 5-FU (7 μg/mL) and resistomycin (1.3 μg/mL). This increase in LDH leakage indicated the potent cytotoxic action of resistomycin, in a dose-dependent way, on PC3 cells (Figure 3).

### 2.3. Resistomycin Suppressed the Antioxidant Response of PC3 Cells

**Figure 4:**
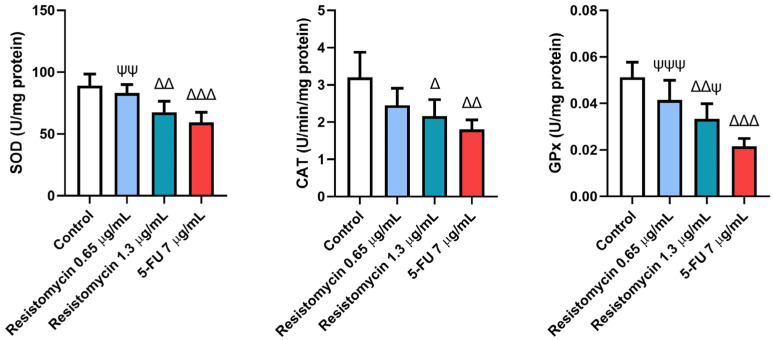
Effects of resistomycin (0.65 or 1.3 µg/mL) or 5-FU treatment (24 h) on the antioxidant enzymatic activities of SOD, CAT, and GPx in PC3 cells. Values represent the mean of three experiments ± SD. ∆: significant with respect to the control (*p* ˂ 0.05). ∆∆: significant with respect to the control (*p* ˂ 0.01). ∆∆∆: significant with respect to the control (*p* ˂ 0.001). Ψ: significant with respect to the 5-FU (*p* ˂ 0.05). ΨΨ: significant with respect to the 5-FU (*p* ˂ 0.01). ΨΨΨ: significant with respect to the 5-FU (*p* ˂ 0.001).

As displayed in Figure 4, the antioxidant enzymatic activities were estimated in treated cells with resistomycin and 5-FU. Substantial declines (*p* < 0.05) were observed in activities of superoxide dismutase (SOD), catalase (CAT), and glutathione peroxidase (GPx) in cells treated with 5-FU (7 μg/mL) and resistomycin (1.3 μg/mL) compared to control cells. However, PC3 cells treated with resistomycin at a dose of 0.65 μg/mL showed a non-significant difference in the activities of these enzymes with respect to the untreated cells. Compared to 5-FU treated cells, there was no different change in SOD or CAT activities when compared with cells treated with resistomycin (1.3 μg/mL). These findings indicate that cells treated with a higher concentration of resistomycin induced an imbalance in the antioxidant defense system. However, the obtained results revealed that 5-FU was able to induce oxidative stress greater than resistomycin in PC3 cells.

### 2.4. Resistomycin Altered the Oxidant Status of PC3 Cells

**Figure 5:**
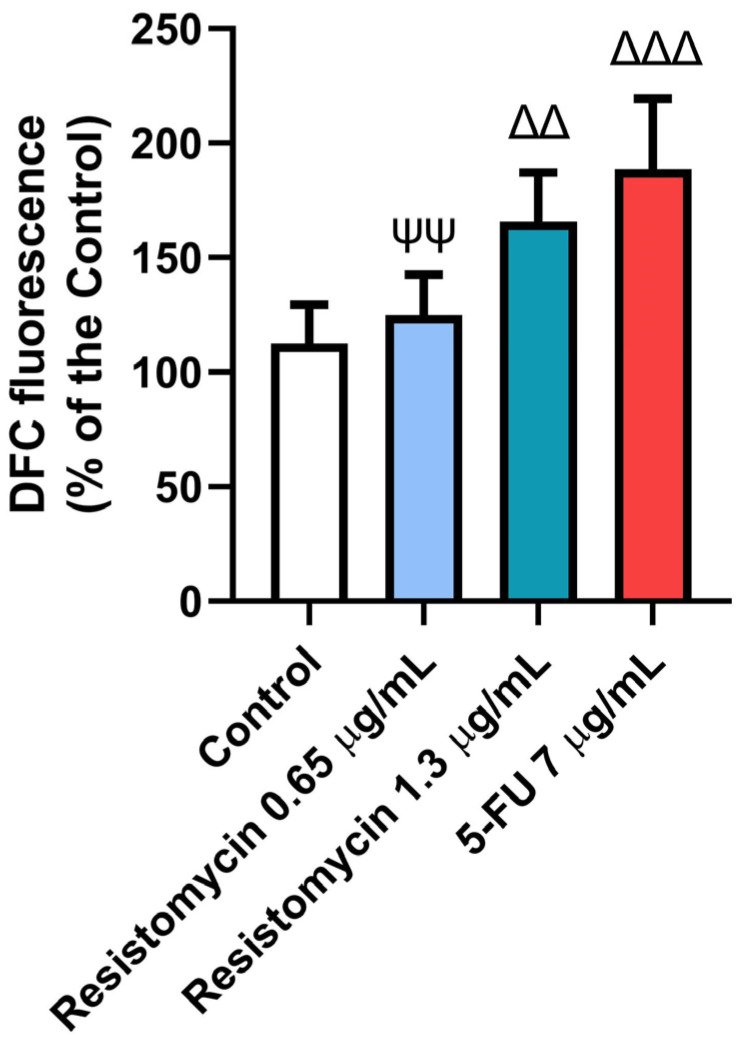
Effects of resistomycin (0.65 or 1.3 µg/mL) or 5-FU treatment (24 h) on ROS in PC3 cells. Values represent the mean of three experiments ± SD. ∆∆: significant with respect to the control (*p* ˂ 0.01). ∆∆∆: significant with respect to the control (*p* ˂ 0.001). ΨΨ: significant with respect to the 5-FU (*p* ˂ 0.01).

**Figure 6:**
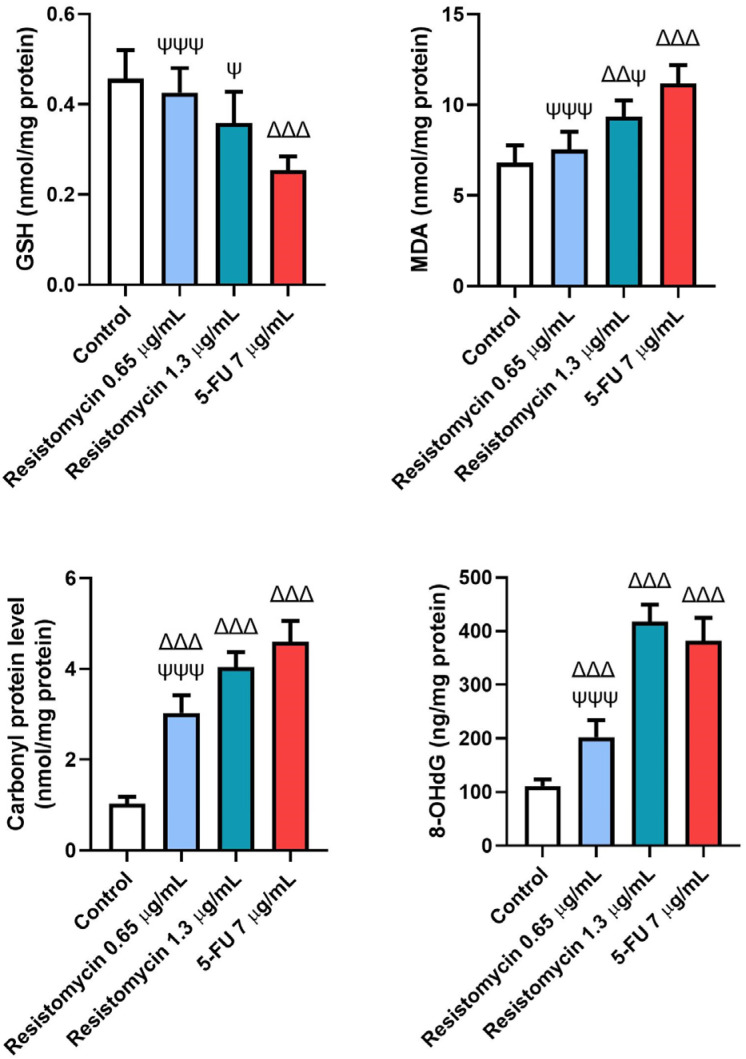
Effects of resistomycin (0.65 or 1.3 µg/mL) or 5-FU treatment (24 h) on the non-enzymatic oxidative stress markers (GSH, MDA, CP, and 8-OHdG) in PC3 cells. Values represent the mean of three experiments ± SD. ∆∆: significant with respect to the control (*p* ˂ 0.01). ∆∆∆: significant with respect to the control (*p* ˂ 0.001). Ψ: significant with respect to the 5-FU (*p* ˂ 0.05). ΨΨΨ: significant with respect to the 5-FU (*p* ˂ 0.001).

The variations in the non-enzymatic markers among the different treatments are shown in Figure 5 and Figure 6. Considerable rises were detected in the measured levels of reactive oxygen species (ROS), malondialdehyde (MDA), carbonyl protein (CP), and 8-hydroxy-2′-deoxyguanosine (8-OHdG) in treated cells with 5-FU (7  μg/mL) and resistomycin (1.3  μg/mL) when compared to control cells. Nevertheless, both doses of resistomycin were not able to decrease glutathione (GSH) content significantly compared to the control. Compared to 5-FU-treated cells, no significant changes in ROS, CP, or 8-OHdG levels were noticed in cells treated with resistomycin at a dose of 1.3  μg/mL. Both doses of resistomycin evoked significantly lower GSH and higher MDA contents with respect to the 5-FU group. Furthermore, the obtained results indicated that 5-FU is able to cause greater oxidative stress than resistomycin at the high dose, as evidenced by the significant change between 5-FU and resistomycin in GSH and MDA levels.

### 2.5. Resistomycin Induced the Levels of Apoptotic Biomarkers in PC3 Cells

**Figure 7:**
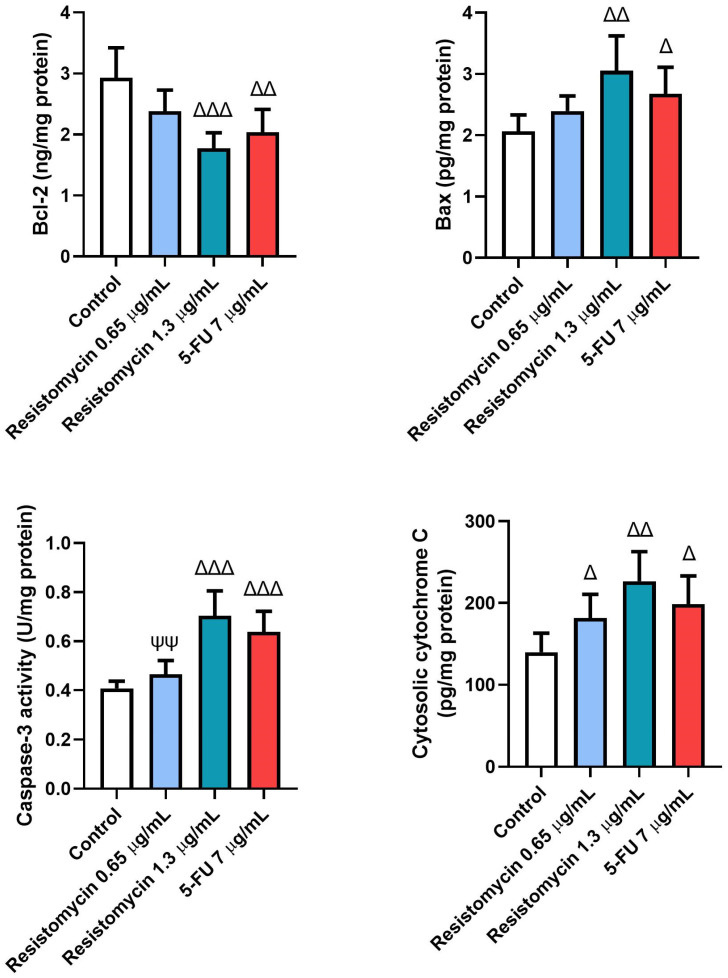
Effects of resistomycin (0.65 or 1.3 µg/mL) or 5-FU treatment (24 h) on the levels of apoptotic markers (Bcl-2, Bax, Cas-3, and cytochrome c) in PC3 cells. Values represent the mean of three experiments ± SD. ∆: significant with respect to the control (*p* ˂ 0.05). ∆∆: significant with respect to the control (*p* ˂ 0.01). ∆∆∆: significant with respect to the control (*p* ˂ 0.001). ΨΨ: significant with respect to the 5-FU (*p* ˂ 0.01).

Remarkable increases (*p* < 0.05) were noticed in the levels of caspase-3 and Bax in PC3 cells subjected to resistomycin (1.3  μg/mL) or 5-FU therapy compared to untreated cells. These pro-apoptotic markers were not significantly changed in cells treated with a lower dose of resistomycin than the control cells. Compared to the treatment with 5-FU, the levels of caspase-3 and Bax did not change in cells that received resistomycin at a concentration of 1.3  μg/mL. Furthermore, noteworthy increases in cytochrome c levels were associated with marked declines (*p* < 0.05) in levels of Bcl-2 in all treated groups with respect to the controls. Compared to the 5-FU treatment, PC3 cells that received resistomycin treatment at both concentrations did not show any changes in cytochrome c levels. Concerning Bcl-2 levels, no remarkable changes were observed in resistomycin (1.3  μg/mL)-treated cells compared to the 5-FU-treated cells (Figure 7). Interestingly, resistomycin at a higher dose was able to induce apoptosis in PC3 cells better than the chemotherapeutic agent, 5-FU.

**Figure 8:**
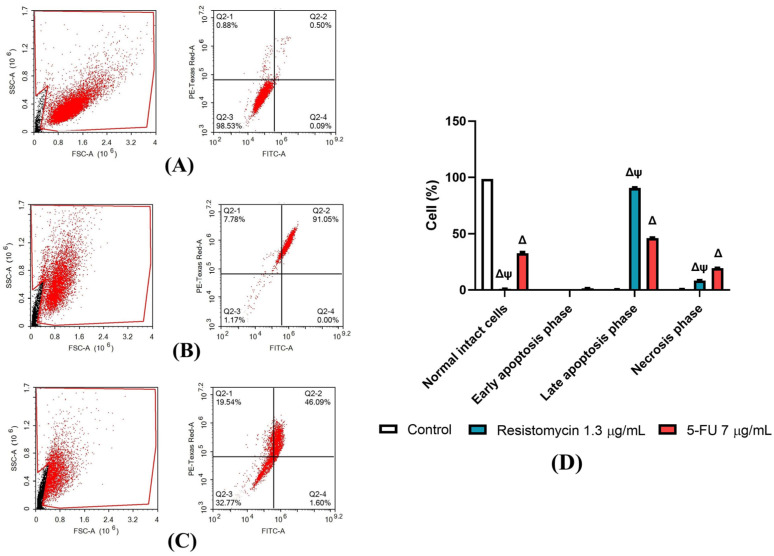
Apoptosis-induced cell death after treatment (24 h) with resistomycin (0.65 or 1.3 µg/mL) or 5-FU in PC3 cell line. Apoptosis-induced cell death was determined through annexin V and PI staining. Percentages of viable, early, late, and necrotic cells are shown when possible. Experiments were performed in triplicate. Values represent the mean of three experiments ± SD. ∆: significant with respect to the control (*p* ˂ 0.05). Ψ: significant with respect to the 5-FU (*p* ˂ 0.05). (**A**): Control, (**B**): Resistomycin, (**C**): 5-FU, (**D**): Histograms showing the different percentages of cell types. Black dots in (**A**–**C**) are 12,000 events acquired and Red dots are the positive FITC and/or PI cells.

To validate the anticancer activity of resistomycin, the apoptosis-induced cell death in PC3 cells that were treated with resistomycin (1.3  μg/mL) or 5-FU (7 μg/mL) were analyzed using annexin V and PI (Figure 8). The data revealed that resistomycin at a concentration of 1.3 µg/mL induced a higher degree of late-stage apoptosis (*p* < 0.05) versus that achieved by 5-FU. In addition, flow cytometric analysis demonstrated that the necrotic cells increased (*p* < 0.05) with resistomycin treatment compared to the control cells; however, the highest necrotic population was observed in 5-FU-treated cells. Resistomycin caused more apoptotic cell death rather than necrosis in PC3 cells. Hence, the data shown clearly suggest that resistomycin has a noteworthy potential to evoke apoptotic cell death in treated PC3 cells.

### 2.6. Resistomycin Promoted the Cell-Cycle-Related Biomarkers in PC3 Cells

**Figure 9:**
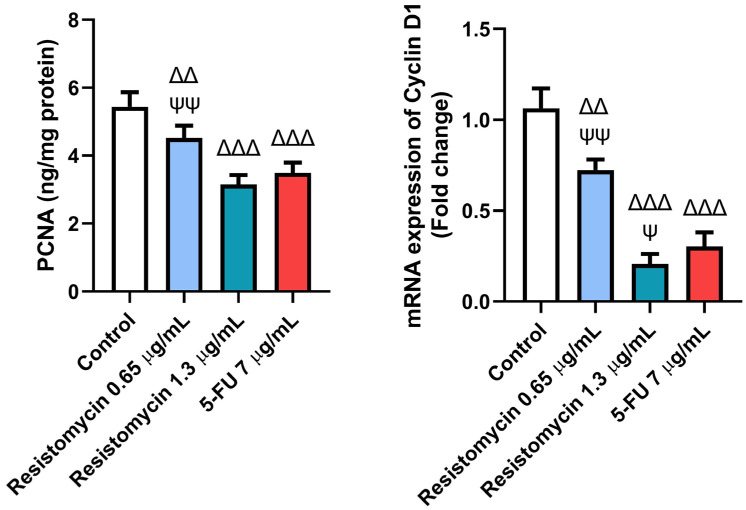
Effects of resistomycin (0.65 or 1.3 µg/mL) or 5-FU treatment (24 h) on the cell-cycle-related markers (PCNA and cyclin D1) in PC3 cells. Values represent the mean of three experiments ± SD. ∆∆: significant with respect to the control (*p* ˂ 0.01). ∆∆∆: significant with respect to the control (*p* ˂ 0.001). Ψ: significant with respect to the 5-FU (*p* ˂ 0.05). ΨΨ: significant with respect to the 5-FU (*p* ˂ 0.01).

To further clarify the mechanisms related to the anticancer activity of resistomycin treatment, the expression of cell-cycle-related markers was performed. Remarkably, resistomycin and 5-FU significantly decreased (*p* ˂ 0.05) the level of proliferating cell nuclear antigen (PCNA) as well as the expression of cyclin D1 in PC-3 cells with respect to the controls. In comparison with 5-FU-treated cells, resistomycin at a concentration of 1.3 µg/mL was able to significantly decrease (*p* ˂ 0.05) the expression of cyclin D1 with a non-significant difference in PCNA levels. These outcomes indicated that resistomycin blocked cell proliferation through the induction of cell cycle arrest of PC3 cells (Figure 9).

## 3. Discussion

Many studies have shown that marine organisms and their derived metabolites exert significant cytotoxic activity against various cancer cell lines [23,24]. In our study, we screened the cytotoxic activity of resistomycin, which is derived from marine actinomycetes, on three cancer cells. MTT assay demonstrated that IC_50_ values for resistomycin and 5-FU in Caco-2 cells were 0.38 μg/mL and 38.47 μg/mL, respectively. Li et al. [23] demonstrated that saquayamycin B1, an angucycline secreted by the marine-derived actinomycete *Streptomyces* sp., has an IC_50_ ranging from 0.18 to 0.84 μM for human colorectal cancer (CRC) cells and an IC_50_ of 1.57 μM for noncancerous normal human hepatocyte (QSG-7701) cells. This indicated that saquayamycin B1 possesses a strong cytotoxicity in cancer cells; however, it has a low cytotoxicity in normal cells. Furthermore, strepyrazinone, another derivative from *Streptomyces* sp. B223, showed cytotoxic activity against HCT-116 (colon cancer cells) and the IC_50_ value of 0.34 µM [25]. In addition, donghaecyclinones, which are derivatives from the *Streptomyces* sp. strain SUD119, displayed cytotoxicity against HCT116 (IC_50_: 8–28.9 µM) and SNU638 (a gastric carcinoma cell line) with an IC_50_ of 9.5–19.6 µM [26].

Moreover, our study unveiled that the IC_50_ values for resistomycin and 5-FU in treated MCF-7 cells were 14.61 μg/mL and 8.03 μg/mL. These findings are in accordance with Qu and collaborators [27], who found significant cytotoxic activity for angucyclines isolated from *Streptomyces* sp. OC1610.4 against breast cancer cells (MCF-7, MDA-MB-231, and BT-474), and the measured IC_50_ values were 0.16–0.67 μM. Similarly, marine mangrove actinobacteria VITGAP173 was reported to exert a promising antitumor effect against MCF-7 cell lines, and the IC_50_ value was calculated as 4.7 μg/mL [28]. Furthermore, resistomycin displayed marked anti-proliferative activity on PC3 cells with an IC_50_ value of 2.63 μg/mL. Likewise, gephyromycin C, a marine-derived actinomycete *Streptomyces* sp. SS13I, has been reported to suppress the growth of PC3 cells with an IC_50_ value of 1.79 ± 0.28 mM [29]. Also, remarkable anti-proliferative activity was noticed by Lin and colleagues [24] in PC3 cells treated with Lu01-M from bacterial marine sediments with IC_50_ values of 1.03 ± 0.31 μg/mL.

Consistent with the findings of the MTT assay, the rate of LDH leakage into the surrounding media was increased in PC3 cells following resistomycin (0.65 and 1.3 μg/mL) treatment, which was noticeably significant from the control group. Similar observations were formerly noticed in PC3 cells treated with potent cytotoxic agents [30,31]. It was previously stated that the extracellular LDH release is an indicator of necrotic cellular death [32]. It can not only reflect cell viability but also give an indication of cell membrane integrity [33]. In accordance with our results, Khalil et al. [34] found that biosynthesized silver nanoparticles from the marine actinobacterium strain *Streptomyces* catenulae M2 induced significant LDH leakage in treated NCM460 and CaCo2 cancer cells. It was also confirmed that the LDH release is closely related to the increased intracellular generation of ROS and the occurrence of the inflammatory response [35]. These findings suggested that resistomycin therapy, in a concentration-dependent manner, can cause cell death in treated PC3 cells by altering the cell membrane integrity and oxidative stress.

Recent growing evidence in tumor investigations has suggested that therapeutic agents exhibit their anticancer effects via their pro-oxidative mechanisms [36,37]. ROS production results in oxidization and damage to cellular DNA, thereby inducing apoptosis and inhibiting cancer [38]. In this investigation, resistomycin therapy triggered oxidative stress in PC3 cells due to a collapse in the activities of antioxidant enzymes and a decline in GSH contents compared to untreated cells. When the ROS levels surpass the ability of prostate cancer cells to eliminate them, this activates cell signaling cascades in the induction of cancer cell death [38]. In addition, this scenario was associated with increased lipid peroxidation and ROS production in treated PC3 cells. As highly reactive compounds, lipid peroxides can enhance the extra generation of ROS or degrade into reactive compounds that are responsible for cytotoxicity. Moreover, these peroxides can subsequently activate the intrinsic and extrinsic apoptotic pathways. In this regard, the intracellular ROS caused a disruption in the mitochondrial membrane potential, which led to the release of cytochrome c, thus inducing apoptosis [39]. Additionally, most of the generated radicals interact with various cellular organic substances, including proteins leading to modified substances. High levels of carbonylated proteins are considered specific indicators for augmented reactive oxygen and nitrogen radicals production in cancer cells [40]. 8-OHdG is another oxidative stress biomarker that represents the end-product of ROS-induced DNA oxidation [41]. A recent investigation has reported high levels of MDA and 8-OHdG in the urine samples of PC patients compared to healthy volunteers [42]. In this regard, Yu and collaborators [43] reported that salinomycin increased the production of ROS, induced lipid peroxidation, and increased the levels of 8-OHdG in PC-3 cells in addition to decreasing the antioxidant enzymes and suppressing the nuclear factor erythroid 2-related factor 2 (Nrf2) signalling pathway. In another study, marine actinomycete Nocardiopsis exhalan-derived metabolite significantly repressed the proliferation of MCF-7 cancer cells in a concentration-dependent manner related to doxorubicin (standard anticancer agent). This was achieved through an imbalance of ROS and by causing oxidative-stress-mediated apoptosis [44]. Therefore, we speculated that resistomycin-induced ROS underlies the cytotoxic effect and cell death of PC3 cells.

The ability of quinones to accept one or two electrons to produce semiquinones and hydroxyquinones, two extremely reactive species, may be the mechanism behind the formation of ROS and the anticancer impact of quinones. The ROS that are produced by molecular oxygen can then oxidize these molecules once more [45]. Quinones can also take part in metabolic processes that convert them to the free radical semiquinone and then to the hydroxyquinone. One-electron reductions are the most common under aerobic conditions, and they produce free radical intermediates [46]. The detoxifying enzyme cytochrome P450 reductase and other flavoprotein enzymes are primarily responsible for carrying them out [44]. An alternate mechanism for the activation involves two-electron reductions, which are then followed by inactivation by glucuronidation, sulfation, or transformation into an alkylating intermediate. This pattern is thought to be the favored one under anaerobic conditions [46], and NQO1 (NADPH: quinone oxidoreductase 1), an enzyme that is highly expressed in cancer tissues [47], is responsible for it.

Furthermore, it was worth speculating that the induction of apoptosis plays an important role in resistomycin-mediated cytotoxic activity in PC3 cells. Apoptosis is a well-organized and orchestrated programmed cellular death that plays a crucial role in maintaining the survival/death balance of cells [48]. The process of antitumor activity always includes apoptosis of the cancer cells. Any imbalance in this process may promote further cancer progress, while boosting apoptotic death may suppress tumor development [49]. In this study, the exposure of PC3 cells to resistomycin (0.65, 1.3 μg/mL) resulted in a dose-dependent increase in several important apoptotic signaling proteins (Bax, caspase-3, and cytochrome-c), suggesting that resistomycin therapy could induce apoptosis in prostate cancer cells. Bcl-2 family proteins are extremely important regulators for the apoptotic signaling pathways. Among these family members, Bax is a pro-apoptotic protein, whereas Bcl-2 is a potent apoptotic suppressor and booth has conflicting functions. After a death signal, the considerable increase in the Bax /Bcl-2 ratio indicates the activation of apoptotic pathways with a subsequent increase in the cells’ vulnerability to die [49]. The imbalance between the Bax and Bcl-2 ratio results in damage to the mitochondrial trans-membrane potential that induces the release of cytochrome-c from the mitochondria into the cytoplasm. Sequentially, cytochrome-c stimulates caspases-3 and initiates the mitochondrial apoptosis [30]. Caspase-3 is a key apoptotic executioner that activates both death receptors and the mitochondrial apoptotic pathways [50]. These results indicate that resistomycin triggered the cell apoptosis in PC3 cells by regulating apoptosis-related molecules. In accordance with our results, *Caulerpa racemosa* extract, a marine seaweed, exerted significant anticancer activity by increasing the expression of Bax and cleaved caspase-3 in HeLa cancer cells [51]. Lin et al. [52] reported that actinomycin V triggered apoptosis in non-small-cell lung carcinoma A549 cells by upregulating both the protein and mRNA expression levels of p53, p21, and Bax. Also, Streptochlorin, a marine *Streptomyces* sp.-derived molecule, evoked notable downregulation of Bcl-2 expression, as well as upregulation of Bax and FasL in human leukemic U937 cells [53].

Furthermore, a study showed that shikonin, one of naphthoquinones, causes the p73 factor to be upregulated in human cervical (HeLa) and breast (MCF-7) cancer cells. This, in turn, results in the downregulation of the anti-apoptotic ICBP90 and the re-expression of p16INK4A, a pro-apoptotic factor and one of p53’s targets [54]. Furthermore, it has been documented that shikonin can activate p53 in response to DNA damage, downregulating Bcl-2 and upregulating Bax, and lowering the expression of cdk4, which causes apoptosis in human malignant melanoma A375-S2 cells [55]. Moreover, a panel of human brain cancer cell lines has shown that plumbagin, another naphthoquinone, can upregulate the expression of p53, induce cell cycle arrest at the G2/M stage, and change the Bax/Bcl-2 ratio [56]. Additionally, in human HepG2 hepatocellular carcinoma cells, it upregulates the expression of the apoptosis markers caspase-3 and -7 [57].

In order to explore more insight into the apoptotic induction mechanism by resistomycin, we analyzed the apoptotic rate in PC3 cells through Annexin V-FITC/PI analysis. This assay depends on the conjugation of annexin V to cell membrane phospholipids when calcium ions are present in the cell. Also, the fluorescent dye PI binds to the cell’s DNA, and it can penetrate the cell membrane of dead cells excluded from healthy cells [58]. In this study, after treatment with resistomycin, a significant increase was noticed in the cells in the late apoptotic phase, indicating the induction of cell death in PC-3 cells. Our results are in harmony with Cho et al. [59], who found that the treatment with manumycin A, a product of *Streptomyces parvulus*, induced a high percentage of apoptotic cells in human oral squamous cell carcinoma. Similarly, alborixin from *Streptomyces scabrisporus* was reported to increase the ratio of the apoptotic population in human colon cancer cells [60].

Another mechanism that is involved in the anti-tumor effect of resistomycin in PC3 cells is cell cycle arrest. Our findings revealed a significant decrease in the levels of PCNA and the mRNA expression of cyclin D1 in PC-3 cells following resistomycin therapy. PCNA is not only a proliferation marker, but it is also related to cell cycle progression, replication, and DNA repair [61]. The suppression of PCNA was found to hinder the growth of tumor cells [62]. Moreover, cyclin D1 proto-oncogene is an important regulator of G1 to S phase progression in many different cell types. Together with its binding partners cyclin-dependent kinase 4 and 6 (CDK4 and CDK6), cyclin D1 forms active complexes that promote cell cycle progression by phosphorylating and inactivating the retinoblastoma protein (RB) [62,63]. On the contrary, PC3 cells that received resistomycin therapy displayed lower PCNA levels and cyclin D1 expression, which indicates that resistomycin is able to restrain cell proliferation and block the cycle at the G1 phase. Likewise, tetracenomycinsare, an antibiotic produced by the *Streptomyces* and *Nocardia* species, induced cell cycle arrest in the lung cancer cells by decreasing the expression levels of cyclin D1 and CDK4 (cyclin-dependent kinase 4) [64]. Similar findings were reported in colorectal cancer cells after being treated with *Streptomyces* sp. 801 [65]. Furthermore, the cytotoxic effect of resistomycin is due to its ability to enact inhibitory action on RNA and protein synthesis with no effect on DNA synthesis. Lin et al. [15] reported that resistomycin and its derivative suppressed tumorigenesis in T24 bladder cancer cells via the downregulation of protein expression sirtuin 1.

## 4. Materials and Methods

### 4.1. Production of Resistomycin

The production of resistomycin was achieved by cultivating *Streptomyces* sp. SP9 on a larger scale (i.e., 6 liters) using Waksman liquid medium [22]. The fermentation process continued for 5 days on a rotary shaker, with the temperature maintained at 28 °C. The culture broth was centrifuged and extracted with organic solvents to obtain a brown crude extract, which was applied to silica gel column chromatography to give four fractions. Fraction II was subjected to further chromatographic purification, leading to the successful isolation of an orange solid.

Resistomycin (1): Orange solid; (-)-ESI-MS *m/z* 375 ([M-H]^−^;(-)-HRESI-MS *m*/*z* 375.0859 (calcd. for C_22_H_15_O_6_, 335.0556); ^1^HNMR ([D6] DMSO, 600 MHz): *δ*_H_ 14.55 (s, 1H, 7-OH), 14.36 (s, 1H, 3-OH), 14.07 (s, 1H, 5-OH), 11.40 (brs, 1H, 10-OH), 7.23 (s, 1H, 11-H), 7.01 (s, 1H, 8-H), 6.34 (s, 1H, 4-H), 2.90 (s, 3H, 9-CH_3_), 1.56 (s, 6H, 1-CH_3_); ^13^CNMR ([D_6_] DMSO, 125 MHz): *δ*_C_ 204.9 (C-2), 183.5 (C-6), 170.7 (C-3), 170.5 (C-5), 167.6 (C-7), 162.1 (C-10), 152.7 (C-11a), 152.1 (C-9), 142.2 (C-11c), 139.1 (C-9b), 128.5 (C-8), 128.4 (C-9a), 118.2 (C-11), 114.2 (C-11b), 107.1 (C-6a), 105.9 (C-5a), 102.1 (C-2a), 99.4 (C-4), 46.1 (C-1), 28.9 (2Me-1), 25.5 (Me-9).

### 4.2. Cell Lines and Culture

The human PC3, DU-145, MCF-7, and Caco-2 cell lines were purchased from VACSERA (Giza, Egypt) and cultured in standard Dulbecco’s Modified Eagle’s Medium (DMEM) (Sigma-Aldrich, Inc., St. Louis, MO, USA) in the presence of 10% heat-inactivated fetal bovine serum (FBS), 4 mM Glutamine, 100 IU/mL streptomycin, and a 100 IU/mL penicillin mixture. Next, the cells were incubated in a humidified incubator (37 °C, 5% CO_2_).

### 4.3. Cytotoxicity Assay

An MTT assay was used for the determination of the cytotoxicity of resistomycin on PC3, DU-145, MCF-7, and Caco-2 cell lines, as reported by Tolosa et al. [66]. In brief, cells were plated at a density of 1 × 10^4^ for each well of a 96-well microtiter plate and exposed to 5-FU and resistomycin. They were incubated at 37 °C in 5% CO_2_ for 24 h. The negative control cells were only treated with DMEM. Consequently, the original medium was replaced from each well, and 100 µL of MTT was added (0.5 mg/mL in PBS, pH 7.2) and incubated at 37 °C for an additional 3 h. The MTT solution was then removed, and 50 µL of DMSO was added to each well until a purple-colored formazan was formed. The absorbance was monitored at 550 nm using a microplate reader, and the optical density was read at a 620 nm wavelength.

### 4.4. LDH Release Assay

The cell cytotoxicity was also monitored through the release of LDH from PC3 cells. Cells were seeded in 96-well plates and allowed to adhere for 24 h. Next, they were incubated for 24 h with different treatments and centrifuged at 10,000× *g* for 10 min. The LDH activity was measured in the supernatant using Cytotoxic 96 ^®^Non-Radioactive Cytotoxicity Assay Kit (Promega, Madison, WI, USA), as per manufacturer’s instructions.

### 4.5. Evaluation of Antioxidant Enzymatic Activities

Superoxide dismutase (SOD) activity in treated PC3 cells was assessed at 480 nm as described by Misra and Fridovich [67]. Catalase (CAT) activity was measured following the protocol described by Aebi [68]. In addition, glutathione peroxidase (GPx) was assessed based on the steps detailed by Paglia and Valentine [69].

### 4.6. Assessment of Oxidative-Stress-Related Markers

#### 4.6.1. Intracellular ROS Measurement

The generation of ROS in PC3 cells was measured using green fluorescence strain 2,7-dichlorofluorescein diacetate (DCFH-DA) [70]. Briefly, PC3 cells were treated with 5-FU and resistomycin for 24 h. Subsequently, cells were washed two times with Hanks’ Balanced Salt solution and incubated in DCFH-DA dissolved in the medium at 37 °C for 30 min. The mean fluorescence intensity of DCF-DA was evaluated using a fluorescence plate reader at 488 and 530 nm, respectively.

#### 4.6.2. GSH Assessment

GSH levels in PC3 cells were estimated using Ellman’s reagent [71]. Cells were mixed with phosphate buffer and DTNB. At the end, the resulting reaction was read at a 412 nm wavelength.

#### 4.6.3. Malondialdehyde Analysis

The lipid peroxidation was estimated in terms of malondialdehyde (MDA) using a colorimetric assay kit [72]. Briefly, PC3 cells were cultured at a density of 1 × 10^4^ cells for each well with the addition of resistomycin and 5-FU to the media. The free MDA reacted with thiobarbituric acid to yield an MDA–TBA adduct. The absorbance of this mixture was measured colorimetrically using the microplate reader at a 532 nm wavelength.

#### 4.6.4. Carbonyl Protein (CP) Assessment

The protein carbonyl content in the cultured PC cells was assessed according to Levine et al. [73]. This method depends on the reaction of oxidized CP groups with 2,4-dinitrophenylhydrazine (2,4-DNPH) to produce dinitrophenyl (DNP) hydrazine. In brief, the treated cell extracts were incubated for one hour with 10-mM 2,4-DNPH. Then, the proteins were precipitated by adding 20% trichloroacetic acid. The precipitate was washed four times with a mixture of ethanol–ethyl acetate (1:1 *v/v*) and then solubilized in 6-M guanidine hydrochloride. The absorbance was measured at 370 nm and calculated in nmols of carbonyl groups/mg of protein.

#### 4.6.5. Assessment of Oxidative DNA Damage

The oxidative damage to DNA in PC3 cells was estimated through the measurement of 8-hydroxy-2′-deoxyguanosine (8-OHdG) using an ELISA kit based on the instructions of the manufacturer. Briefly, 100 μL of treated cell lysates was added to an antibody pre-coated microtiter plate and left to be incubated at 37 °C for 1 h prior to adding 8-OHdG’s substrate. This mix was left at room temperature for a period of 15 min of incubation, and then a 50 μL stop solution was added. The absorbance of the developed color was assessed at 450 nm, and the results were expressed in ng/mg protein.

### 4.7. Estimation of Apoptosis-Related Biomarkers

Pro-apoptotic markers (cytochrome c, Bax, and caspase-3) were assayed in PC3 cells using ELISA kits from Cusabio, Wuhan, China following the manufacturer’s instructions. The catalogue Number for Cytochrome c is CSB-EL006328RA, Bax: CSB-EL002573RA, and caspase-3: CSB-E08857r. In addition, the anti-apoptotic marker, Bcl-2, was also assessed using CSB-E08854r Kits according to the protocol information.

### 4.8. Apoptosis Analysis through Annexin V/Propidium Iodide Assay

In our study, the impact of resistomycin on the cell death rate was assessed using the double staining method of annexin V and PI in treated PC3 cells. The effect of 5-FU and resistomycin (1.3 μg/mL) on the apoptotic rate was investigated using an Annexin V-FITC/PI (C1062, Beyotime, Shanghai, China) apoptosis detection kit. After trypsin digestion without ethylene diamine tetraacetic acid, the cells were collected through centrifugation and re-suspended. Annexin V-FITC and propidium iodide were added to the cell suspension in an ice bath. The staining of nuclei was analyzed using a FACS flow cytometer (Becton Dickinson, San Jose, CA, USA). Resistomycin at a concentration of 0.65 µg/mL was omitted from this experiment, as the obtained results demonstrated that resistomycin at a low dose failed to change Bax and caspase-3 significantly.

### 4.9. Analysis of Cell-Cycle-Related Markers

The levels of proliferating cell nuclear antigen (PCNA) in treated PC3 cells were assessed using ELISA kits (ab196270, Abcam, Cambridge, UK) based on the manufacturer’s information. Furthermore, a quantitative RT-PCR technique was utilized for the measurement of the mRNA levels of cyclin D1 in PC3 cells. The extraction of total RNA was performed from freshly isolated cells using TRIzol reagent (Qiagen, Germantown, MD, USA), following the manufacturer’s instruction. Next, RNA concentrations were measured using nanodrop, and cDNA synthesis was performed using the RevertAid™ H Minus Reverse Transcriptase kit (Fermentas, ThermoFisher Scientific Inc., Mississauga, ON, Canada), following the manufacturer’s information. The SYBR green PCR kit (Qiagen, Hilden, Germany) was used to measure the relative levels of mRNA. Quantitative PCR was performed in triplicate on the ViiATM 7 PCR system (Applied Biosystems, Foster City, CA, USA). The relative levels of mRNA were calculated using the 2^−ΔΔCt^ method and normalized to the mRNA level of β-actin (housekeeping gene). The primer sequences are presented in Table 1.

### 4.10. Statistical Analysis

The IC_50_ values were obtained using the Quest Graph™ IC_50_ Calculator, AAT Bioquest, Inc., Pleasanton, CA, USA (https://www.aatbio.com/tools/ic50-calculator, accessed on 28 October 2023) online resource. All data were statistically analyzed using one-way ANOVA to assess the significant difference between differently treated groups followed by Tukey’s post hoc test using the software GraphPad Prism version 8 (San Diego, CA, USA). The mean values of the MTT assay results between the control and the treatment were compared using paired samples Student’s *t*-test. Data were displayed as the mean ± standard deviation (SD). A *p*-value of less than 0.05 was considered significantly different.

## 5. Conclusions

Taken together, our study demonstrated the therapeutic efficacy of resistomycin at two different concentrations for the suppression of prostate cancer cell growth. This action was achieved via the generation of excess ROS with subsequent oxidative stress in tumor cells. In addition, resistomycin treatment significantly induced apoptotic cell death and cell cycle arrest in PC3 cells. This study illustrated the therapeutic capacity of resistomycin as a marine-derived secondary metabolite for being potentially applied for PC treatment. This may initiate a new therapeutic paradigm in the field of oncology or beyond, for other human diseases.

## Figures and Tables

**Table 1:** The sequences of the qPCR primers.

Gene	Accession Number	Forward (5′-3′)	Reverse (5′-3′)
Cyclin D1	NM_053056.3	GAGGCGGAGGAGAACAAACA	GGAGGGCGGATTGGAAATGA
β-actin	NM_001101.5	AGCCTCGCCTTTGCCG	CGCGGCGATATCATCATCCA

## Data Availability

All of the relevant data are within the paper.

## Supplementary Materials

The following supporting information can be downloaded at: https://www.mdpi.com/article/10.3390/molecules28237871/s1, Figure S1: (-)-ESI-MS of resistomycin. Figure S2: UV spectra of resistomycin in MeOH. Figure S3: ^1^H NMR spectrum (DMSO-d_6_,600 MHz) of resistomycin. Figure S4: ^13^C NMR spectrum (DMSO-d_6_, 150 MHz) of resistomycin. Figure S5: Effects of different concentrations of resistomycin or 5-FU treatment (24 h) on PC3, DU-145, MCF-7 and Caco-2 cell lines. The graphs and equations generated using Quest Graph™ IC50 Calculator. Figure S6: Effects of different concentrations of resistomycin or 5-FU treatment (24 h) on PC3, DU-145, MCF-7 and Caco-2 cell lines.
