# Supplementary material for: Resistomycin Suppresses Prostate Cancer Cell Growth by Instigating Oxidative Stress, Mitochondrial Apoptosis, and Cell Cycle Arrest
Source: Molecules. 2023 Nov 30;28(23):7871. doi: 10.3390/molecules28237871 (PMC10708360; doi:10.3390/molecules28237871)
Supplement: Supplementary file 1 [file molecules-28-07871-s001.zip › molecules-2716418-supplementary.pdf]

Supporting Information

Resistomycin Suppresses Prostate Cancer Cell Growth by Instigating Oxidative Stress, Mitochondrial Apoptosis, and Cell Cycle Arrest

Abeer S. Aloufi [1], Ola A. Habotta [2], Mohamed S. Abdelfattah [3], Marina N. Habib [3], Mohamed M. Omran [3], Sally A. Ali [4], Ahmed E. Abdel Moneim [5,*], Shereen M. Korany [1], Aisha M. Alrajhi [1]

1 Department of Biology, College of Science, Princess Nourah bint Abdulrahman University, P.O. Box 84428, Riyadh 11671, Saudi Arabia; asaloufi@pnu.edu.sa (A.S.A.); smkorany@pnu.edu.sa (S.M.K.); amalrajhi@pnu.edu.sa (A.M.A.)
2 Department of Forensic Medicine and Toxicology, Faculty of Veterinary Medicine, Mansoura University, Mansoura 35516, Egypt; ola_ali@mans.edu.eg
3 Chemistry Department, Faculty of Science, Helwan University, Cairo 11795, Egypt; mabdelfattah@science.helwan.edu.eg (M.S.A.); marinanabil2104@gmail.com (M.N.H.)
4 Botany and Microbiology Department, Faculty of Science, Helwan University, Cairo 11795, Egypt; sally_ali@science.helwan.edu.eg
5 Zoology and Entomology Department, Faculty of Science, Helwan University, Cairo 11795, Egypt

* Correspondence: ahmed_abdelmoneim@science.helwan.edu.eg

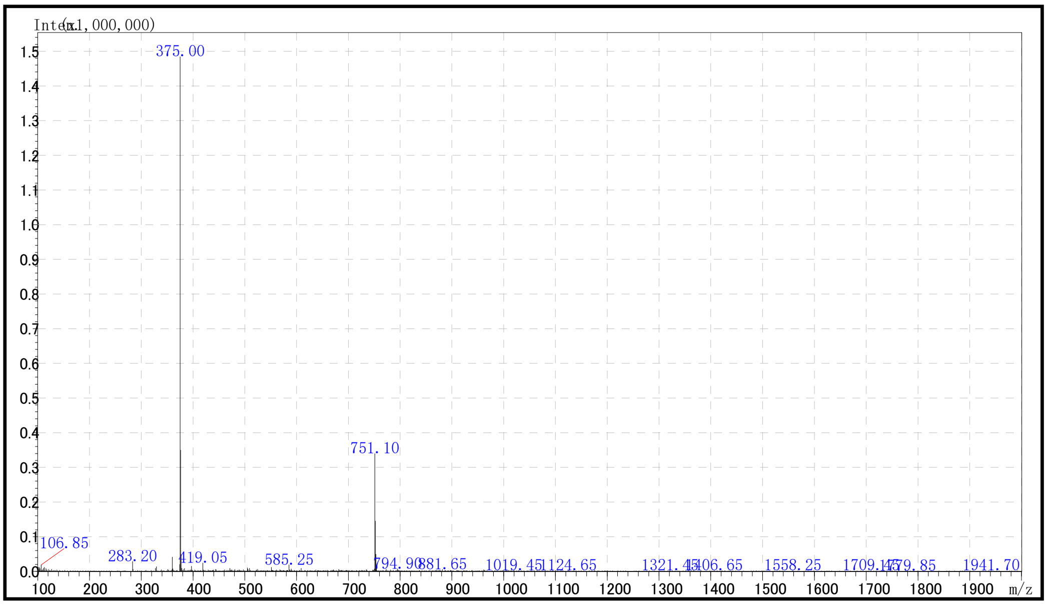

Figure S1: (-)-ESI-MS of resistomycin

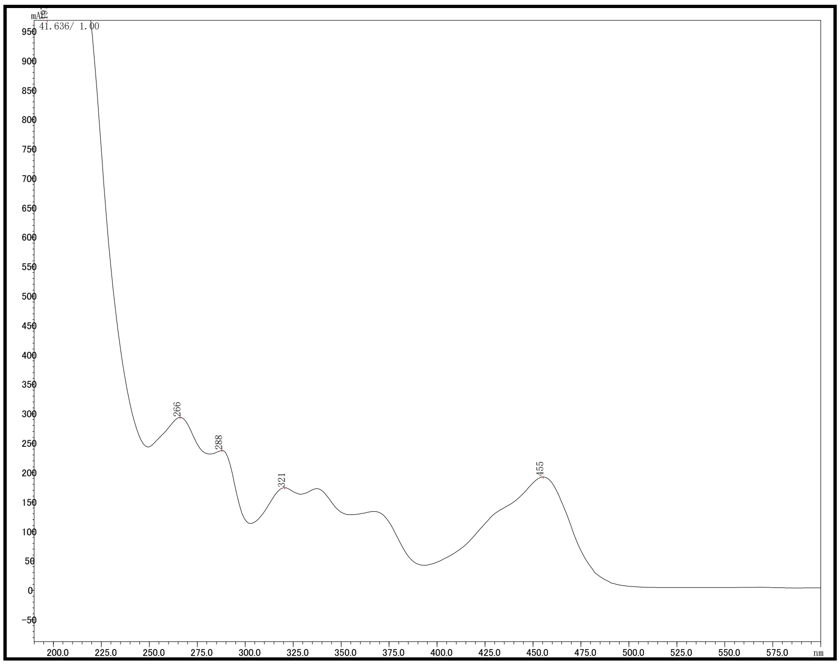

Figure S2: UV spectra of resistomycin in MeOH

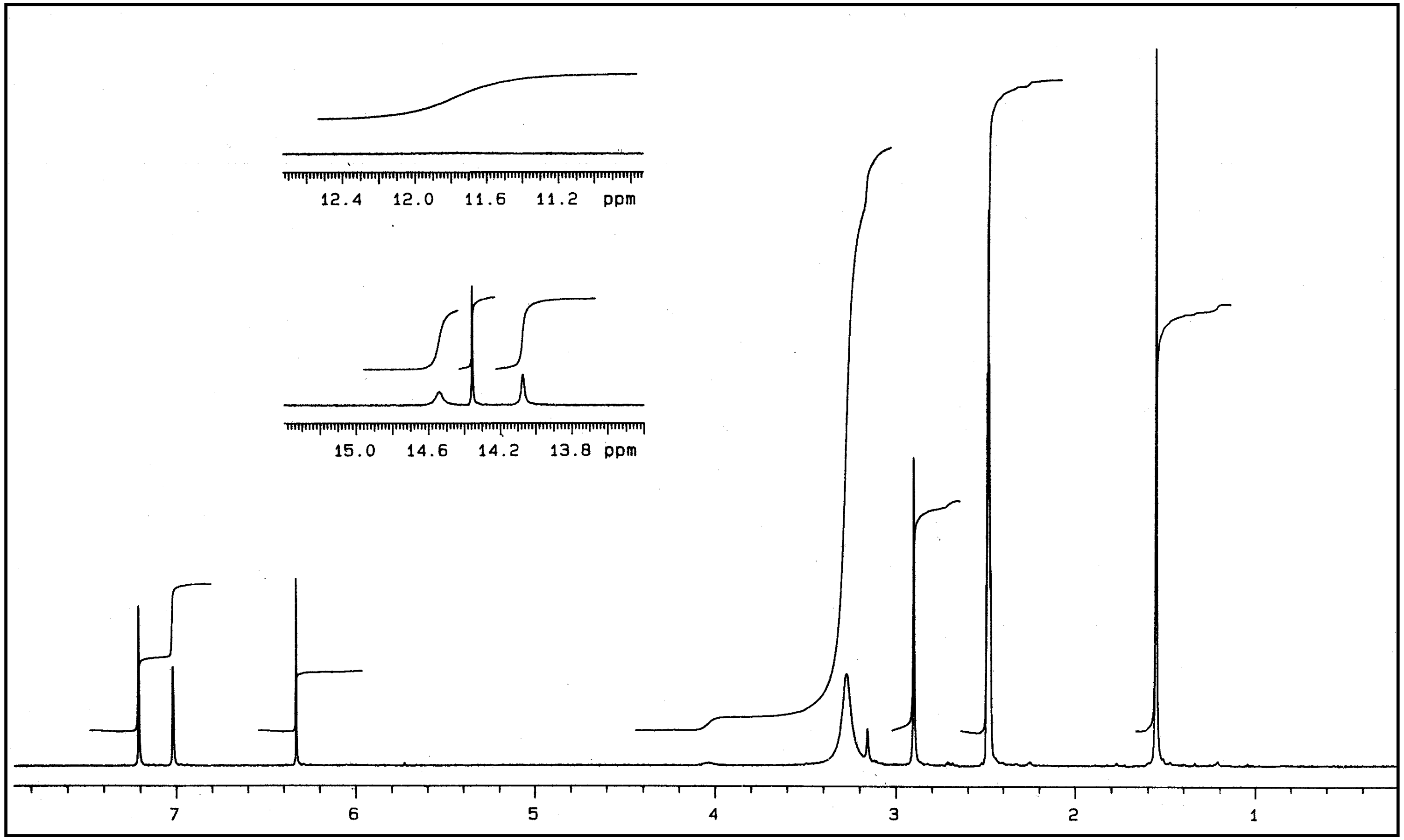

Figure S3: ^{1}H NMR spectrum (DMSO-d$_6$,600 MHz) of resistomycin

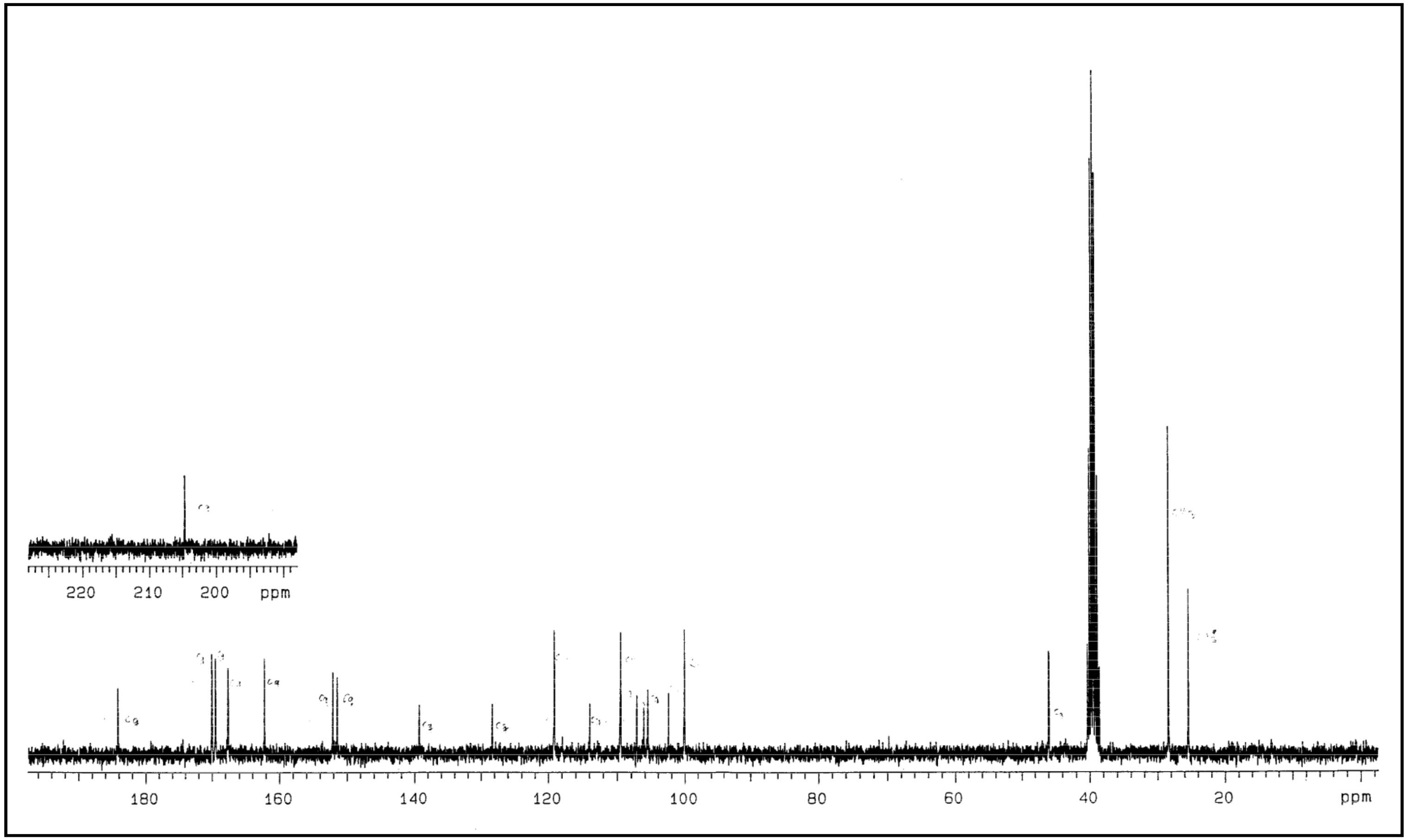

Figure S4: ^{13}C NMR spectrum (DMSO-d_6, 150 MHz) of resistomycin

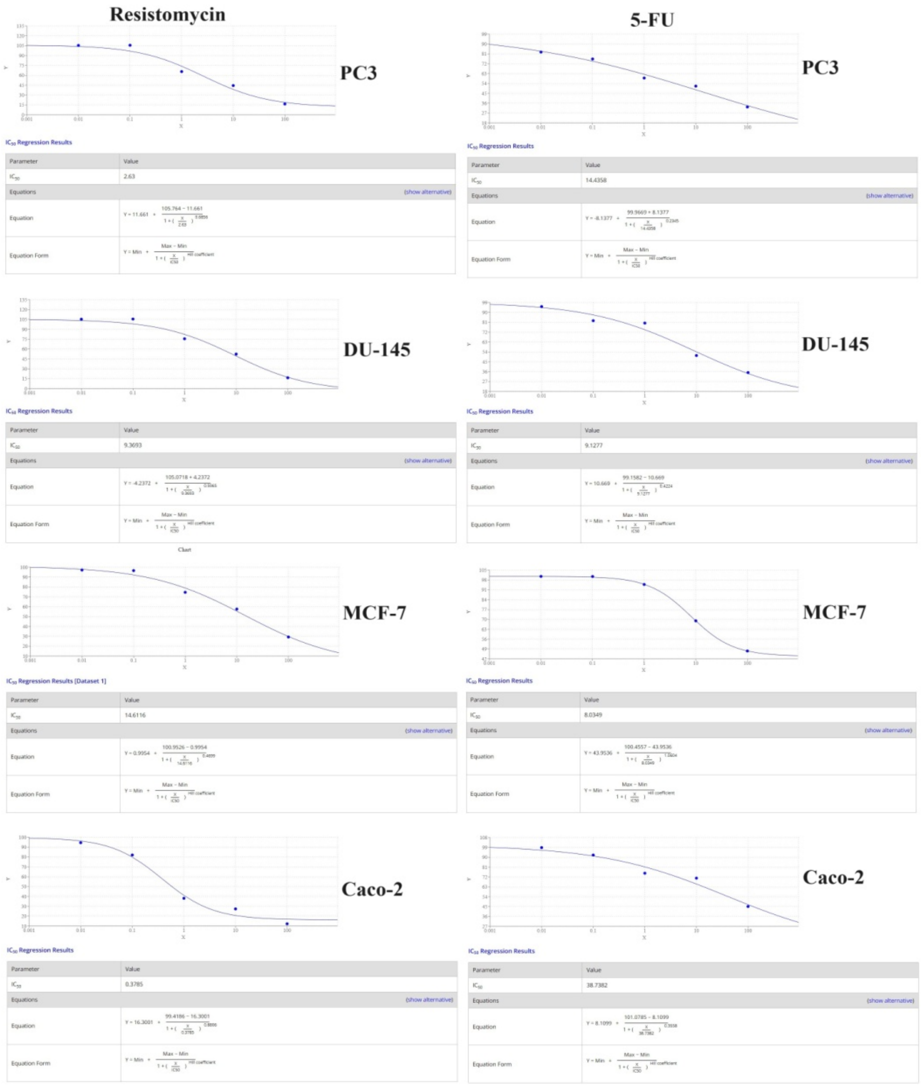

Figure S5: Effects of different concentrations of resistomycin or 5-FU treatment (24 h) on PC3, DU-145, MCF-7 and Caco-2 cell lines. The graphs and equations generated using Quest Graph™ IC50 Calculator." *AAT Bioquest, Inc.*, 28 Oct. 2023, https://www.aatbio.com/tools/ic50-calculator.

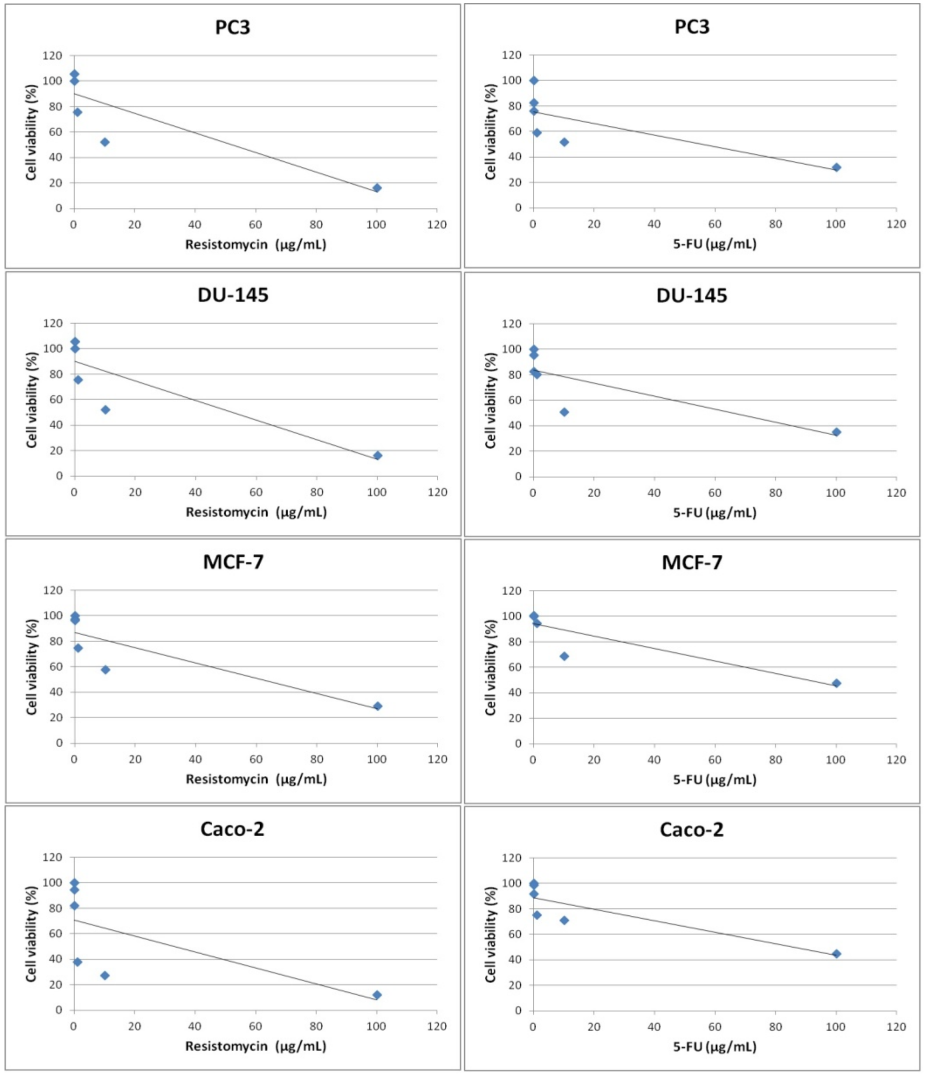

Figure S6: Effects of different concentrations of resistomycin or 5-FU treatment (24 h) on PC3, DU-145, MCF-7 and Caco-2 cell lines. The graphs generated using Microsoft Corporation. (2018). Microsoft Excel. Retrieved from https://office.microsoft.com/excel.
